# Digital literacy and subjective wellbeing of young people: a mixed-methods approach based on SEM and fsQCA

**DOI:** 10.3389/fpsyg.2026.1818046

**Published:** 2026-04-30

**Authors:** Yajun Wang, Minting Lai, Zhuo Liu

**Affiliations:** 1 School of Energy and Chemical Engineering, Luoyang Institute of Science and Technology, Luoyang, China; 2 School of Economics, Fujian Normal University, Fuzhou, China

**Keywords:** digital literacy, self-efficacy, social support, subjective wellbeing, young people

## Abstract

**Introduction:**

As digital natives and intensive users of digital technologies, young people's digital literacy has become a critical determinant of their subjective wellbeing. Based on the COR theory, this study explores the impact of digital literacy on young people's subjective wellbeing and its underlying mechanisms.

**Methods:**

This study adopts a comprehensive methodological framework integrating Structural Equation Modeling (SEM) and fuzzy-set Qualitative Comparative Analysis (fsQCA). SEM is employed to examine the direct and mediating relationships among digital literacy, social support, self-efficacy, and subjective wellbeing within a resource-based framework. Meanwhile, fsQCA is used to explore multiple configurational pathways through which different combinations of resources contribute to high levels of subjective wellbeing.

**Results:**

Results revealed three key findings:(1) Digital literacy is positively associated with young people's subjective wellbeing and demonstrates a resource accumulation and upgrading pattern, evolving from basic operational skills to higher-order cognitive and professional competencies. (2) Social support and self-efficacy function both as independent mediators and as sequential resources in a resource gain chain: digital literacy → social support → self-efficacy → subjective wellbeing. (3) The fsQCA reveals three functionally equivalent configuration pathways: S1, a “security–professional–support pathway”; S2, a “cognitive–operational–social collaboration pathway”; and S3, a “comprehensive capability–driven pathway.”

**Discussion:**

These findings provide empirical support for COR theory by demonstrating how digital literacy, as a foundational personal resource, facilitates the accumulation and interaction of social and psychological resources to enhance subjective wellbeing. The study highlights the importance of strengthening digital literacy education, promoting equitable access to digital resources, fostering youth digital engagement, and improving platform governance and privacy protection. Such efforts can help build sustainable resource gain cycles and maximize the wellbeing benefits of digital literacy among young people.

## 1 Introduction

The 21st century is defined by networks and information technologies. Digital technologies such as 5G, big data, and artificial intelligence have been integrated into people's daily lives, altering traditional ways of working and studying, and they have also become an essential part of people's social lives (Shanmugasundaram and Tamilarasu, 2023). In the digital era, digital literacy has become a fundamental and crucial skill needed to adapt to the accelerated digital society (Castillo de Mesa et al., 2020). Digital literacy not only facilitates the effective use of digital tools and information resources—thereby enhancing knowledge acquisition and accelerating technology diffusion—but also fundamentally reshapes production modes and organizational structures (Sharma et al., 2016; Marangell and Randall, 2025). As the digital economy deepens, improving digital literacy at the population level has become both a strategic imperative for human development and a crucial measure for narrowing the digital divide (Aydin, 2021).

As digital technologies increasingly permeate everyday life, individuals are not merely users of digital tools but also active participants in digitally mediated social and psychological environments. In this sense, digital literacy may influence not only individuals' access to opportunities but also how they experience, interpret, and evaluate their lives (Choung et al., 2023). Subjective well-being is an interdisciplinary concept spanning psychology and economics that reflects individuals' self-assessments of their quality of life. Subjective well-being is generally understood as an individual's subjective evaluation of their overall quality of life based on self-defined life goals or standards, encompassing satisfaction with life, as well as judgments of positive and negative emotions (Diener et al., 2018). Although China has experienced sustained economic growth since the reform and opening-up period, national subjective wellbeing has not risen correspondingly (Yang et al., 2022a). According to the Huang et al. (2023), China ranked 72nd among 146 countries and regions, indicating a mid-to-lower level of wellbeing. Identifying the determinants of residents' subjective wellbeing and enhancing public welfare therefore remain important areas of inquiry. This issue is especially salient for young people, whose growth and development are deeply intertwined with the Internet. For young people, digital literacy is both a foundational capability for navigating the digital era and a critical bridge connecting mental health and social adaptation (Narros-González et al., 2025). It determines whether young people can effectively manage information and social resources in increasingly complex digital environments and achieve holistic development and improved wellbeing. In this context, examining whether digital literacy can serve as a positive force—an “enhancer”—of young people's subjective wellbeing is of considerable importance.

Grounded in Conservation of Resources (COR) theory, this study integrates Structural Equation Modeling (SEM) and fuzzy-set Qualitative Comparative Analysis (fsQCA) to examine how digital literacy, as a critical personal resource, shapes young people's subjective wellbeing through resource gain processes. By combining mediation analysis with configurational approaches, the study not only identifies the indirect pathways through which digital literacy operates via social and psychological resources but also uncovers multiple resource configurations leading to high wellbeing, thereby offering a comprehensive understanding of resource gain dynamics and actionable insights for enhancing youth wellbeing in the digital era. This study seeks to answer the following questions: (1) What structural relationship exists between digital literacy and young people's subjective wellbeing? Do different dimensions of digital literacy—digital cognitive literacy, digital operational literacy, digital safety literacy, and digital professional literacy—exert heterogeneous effects on subjective wellbeing? (2) Do social support and self-efficacy mediate the relationship between digital literacy and young people's subjective wellbeing? Do they operate as a sequential mechanism in this relationship? (3) From a configurational perspective, how do different combinations of digital literacy, social support, and self-efficacy jointly generate multiple pathways leading to high (or low) levels of subjective wellbeing among young people?

## 2 Literature review

### 2.1 Digital Literacy

With the deep embedding of digital technologies and the rapid expansion of the platform economy, digital literacy has evolved from a peripheral competence into a foundational determinant of individual development and social stratification (De Vries et al., 2025). Early studies primarily conceptualized it as operational proficiency in information technologies, focusing on individuals' mastery of computers and internet-based tools (Gilster, 1997). As the informational environment has grown increasingly complex, this concept has been progressively broadened to encompass multidimensional capacities, including information access, evaluation, integration, and creation, as well as higher-order dimensions such as critical thinking and digital ethical awareness (Guo and Ogbodo, 2026). Accordingly, digital literacy can no longer be reduced to a set of technical skills; rather, it constitutes a comprehensive capability system embedded within social contexts, whose core lies in individuals' capacity to make effective decisions and take purposeful action in complex information environments.

At the empirical level, digital literacy has been shown to exert significant effects on educational attainment, employment opportunities, and social participation (Li, 2025; Zhang, 2025). On the one hand, higher levels of digital literacy reduce the costs of information acquisition and enhance the efficiency of resource allocation, thereby conferring advantages in knowledge acquisition and opportunity recognition (Chen and Xiao, 2024). On the other hand, by shaping individuals' modes of information processing and behavioral patterns, digital literacy profoundly influences their pathways of engagement in social interactions and institutional processes (Brown, 2026). In particular, under conditions of deepening platformization and algorithmic governance, the ability to effectively comprehend and navigate digital environments has become a critical factor in determining individual life trajectories (Abah, 2025).

Notably, digital literacy among youth exhibits a distinctive pattern characterized by the coexistence of access advantages and capability stratification (Martini and Sgambato, 2025). As “digital natives,” young people possess inherent advantages in device usage and network access; however, substantial disparities persist in their abilities related to information evaluation, deep processing, and rational judgment (Anderson, 2019). This layered differentiation, spanning from surface-level access to deeper cognitive competencies, renders digital literacy a key explanatory variable for intra-cohort heterogeneity among youth (Kreuder et al., 2024). Nevertheless, the extant literature has predominantly emphasized the economic returns and human capital attributes of digital literacy, while paying comparatively limited attention to its psychological and welfare implications. In particular, systematic efforts to incorporate digital literacy into analytical frameworks of subjective experience remain scarce.

### 2.2 Subjective wellbeing

Subjective wellbeing, as a central dimension for assessing individuals' quality of life, is typically understood as comprising life satisfaction and affective experiences (Nima Al et al., 2024). Its formation is shaped not only by objective resource constraints but also by individuals' cognitive evaluations and processes of psychological adjustment (Rzeszutek et al., 2025). Traditional research has primarily approached this construct from the perspective of resource endowments—such as income, education, and health—emphasizing the foundational role of material conditions in supporting wellbeing (Burova, 2025), while also underscoring the importance of social capital and institutional contexts in shaping individual wellbeing (Deng et al., 2018). Subsequent studies have incorporated psychological perspectives, highlighting the mediating roles of cognitive mechanisms such as self-efficacy, perceived control, and social comparison, thereby establishing an analytical framework that links resources, cognition, and wellbeing (Beier et al., 2023).

In the context of digitalization, the subjective wellbeing of youth exhibits an emerging and distinct generative logic. With the widespread diffusion of the internet and mobile technologies, information acquisition, social interaction, and self-expression have become increasingly mediated by digital platforms, transforming the digital environment from an external condition into a core determinant of lived experience (Mayen et al., 2025). On the one hand, digital technologies expand informational boundaries and social networks (Yasmine et al., 2025), providing young individuals with more diverse opportunities for development and expression, thereby enhancing their sense of participation and perceived gains (Martin-Barrado and Gomez-Baya, 2025). On the other hand, phenomena such as information overload, algorithmic curation, and online comparison mechanisms may intensify feelings of anxiety, loneliness, and uncertainty, exerting adverse effects on subjective wellbeing (Teng and Cho, 2025).

Although existing studies have begun to examine the impact of digital environments on subjective wellbeing, they have largely relied on external indicators—such as frequency of media use or time spent online—as primary analytical entry points, with limited attention to heterogeneity in individuals' capabilities within digital contexts (Ding and Zhang, 2025). In fact, differences in how individuals process information, select content, and engage in interactions may constitute a crucial mechanism underlying divergence in their wellbeing outcomes (McKenzie and Feng, 2026).

Taken together, while prior research has generated substantial insights into digital literacy and subjective wellbeing, it remains fragmented and lacks an integrated analytical framework. In particular, insufficient attention has been paid to capability heterogeneity in digital contexts, with limited efforts to explain variations in subjective wellbeing from the perspective of digital literacy as an endogenous competence. Moreover, studies on youth—despite recognizing them as primary users of digital technologies—are largely descriptive and fall short of systematically unpacking underlying mechanisms. Existing analyses also tend to rely on external indicators such as usage frequency, while overlooking key mediating processes, including information processing and cognitive evaluation, and lacking coherent theoretical integration across mechanisms. Addressing these gaps, this study focuses on youth and incorporates digital literacy into the analysis of subjective wellbeing, aiming to advance a more parsimonious and explanatory framework for understanding wellbeing formation in the digital age.

## 3 Theoretical analysis and hypothesis

**Figure 1:**
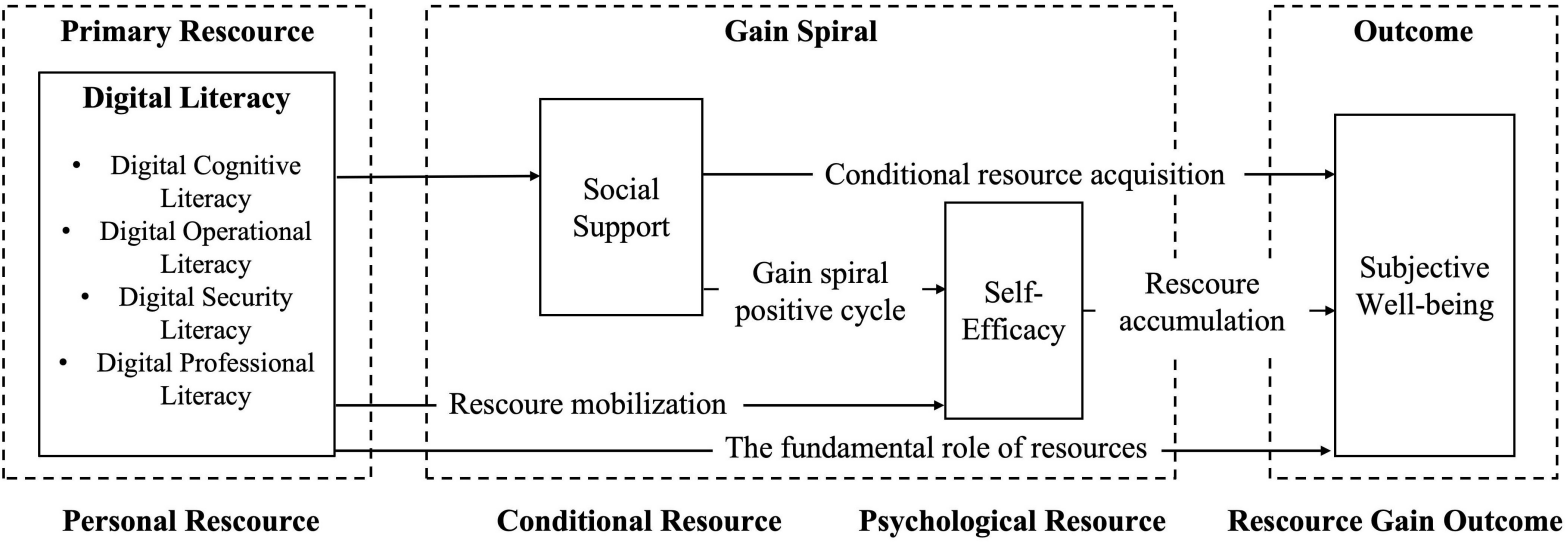
The theoretical framework.

Grounded in Conservation of Resources Theory (COR), individuals are posited to strive to acquire, retain, and protect valued resources (Hobfoll, 1989). The accumulation of such resources enhances adaptive capacity and psychological wellbeing, whereas resource scarcity or loss precipitates stress and a decline in wellbeing (Li et al., 2026). Within the context of a digitalized society, digital literacy has emerged as an instrumental asset enabling young individuals to access information, expand social networks, and realize self-worth (Caroline et al., 2025). Social support and self-efficacy represent, respectively, external social resources and internal psychological resources (Shi et al., 2025). Together, these elements constitute a multi-layered resource system that influences youths' subjective wellbeing through resource gain processes. Drawing on COR theory, this study conceptualizes digital literacy as a primary resource, with social support and self-efficacy as derivative resources, and constructs a resource gain chain to systematically elucidate the mechanisms underlying the formation of subjective wellbeing among young people. The specific research framework is shown in Figure 1.

### 3.1 Digital literacy and subjective wellbeing

In the digital era, digital literacy represents a distinctive, composite core capability resource among youth. It is not a unidimensional construct but rather an integrated system encompassing multiple dimensions, including digital cognition, technical operation, risk mitigation, and professional application (Tinmaz et al., 2022). These differentiated dimensions exert systematic effects on youths' subjective wellbeing through heterogeneous resource transmission mechanisms.

At the foundational level, digital operational literacy reflects individuals' capacity to effectively utilize digital tools and constitutes the foundational level of digital literacy, serving as the logical starting point for youth to acquire digital resources (Ng, 2012). From the perspective of COR Theory, its core value lies in reducing the temporal and cognitive costs associated with information search, interpersonal communication, and task execution, thereby enhancing efficiency in accessing digital environments and obtaining informational and social resources (Meier and Bol, 2025). Such improvements in resource acquisition efficiency help alleviate psychological stress and anxiety arising from resource scarcity and constrained access channels, while simultaneously establishing a solid foundation for subsequent resource accumulation and transformation, consistent with the COR proposition that initial resource accumulation is essential for mitigating resource loss and promoting wellbeing (Buckingham et al., 2023).

Building on this foundation, digital cognitive literacy advances to the dimension of cognitive deepening, which encompasses individuals' abilities to filter, evaluate, critically interpret, integrate, and logically process vast amounts of digital information (Georgopoulou et al., 2025). This stage signifies a transition from passive resource acquisition to active resource utilization. In the digital age, phenomena such as information overload, misinformation, and cognitive bias are likely to induce cognitive dissonance and psychological uncertainty among youth, resulting in latent depletion of psychological resources (Carlsson, 2026). By contrast, advanced digital cognitive capabilities enable individuals to navigate informational noise effectively, enhance interpretive control over both digital environments and external realities, and establish stable cognitive resource reserves. Within the COR framework, cognitive resources—as a critical form of internal resource—not only reduce potential psychological resource loss but also improve the allocation and utilization efficiency of external resources. Consequently, they strengthen psychological security and emotional stability, thereby providing a cognitive foundation for enhanced subjective wellbeing.

At a more advanced level, digital literacy is embodied in professional transformation capability, through which individuals embed digital operational and cognitive competencies into domains such as learning, work, social interaction, and innovative practice, thereby converting digital capabilities into human capital, social capital, and tangible value creation (Lu et al., 2026). From this perspective, digital literacy transcends its instrumental and cognitive dimensions to become a core productive resource that enables youth to accumulate capital, build competitive advantage, and realize self-worth. COR theory emphasizes that recursive resource appreciation and spiral gains constitute the fundamental mechanism for sustained wellbeing enhancement. The professional transformation of digital literacy serves as a critical driver of this process. Through the practical application of digital capabilities, youth not only obtain tangible returns—such as academic advancement and career development—but also reinforce self-identity, sense of achievement, and self-efficacy in the process of value creation, thereby promoting subjective wellbeing from both psychological and material dimensions (Deja et al., 2024).

Moreover, digital safety literacy functions as a cross-cutting protective dimension across all facets of digital literacy, consistently fulfilling the roles of resource preservation and risk mitigation. This function aligns closely with the COR principle of prioritizing resource protection (Dev et al., 2024). Whether manifested as technical risks at the stage of tool use, informational risks during cognitive processing, or privacy risks in the phase of professional transformation, digital safety literacy enables youth to effectively identify, prevent, and respond to diverse digital threats (Cowling et al., 2025). It reduces the probability of resource loss and ensures the continuity and stability of resource accumulation, transformation, and appreciation processes. The first hypothesis of the study is as follows:

H1: Digital literacy is positively associated with young people's subjective wellbeing.
H1a: Digital cognitive literacy is positively associated with young people's subjective wellbeing.
H1b: Digital operational literacy is positively associated with young people's subjective wellbeing.
H1c: Digital safety literacy is positively associated with young people's subjective wellbeing.
H1d: Digital professional literacy is positively associated with young people's subjective wellbeing.

### 3.2 The mediating role of self-efficacy

Self-efficacy, as conceptualized by Bandura (1977), refers to individuals' beliefs in their capabilities to organize and execute the actions required to manage prospective situations or accomplish specific tasks. Within the analytical framework of COR Theory, self-efficacy constitutes a pivotal psychological resource that functions not only as an outcome of resource accumulation but also as a motivational driver for subsequent resource acquisition (Yan and Hu, 2025).

The effect of digital literacy on self-efficacy can be understood through two primary mechanisms. First, the competence validation mechanism: digital literacy entails individuals' proficiency in operating digital tools, solving problems, and critically evaluating information within digital contexts. These repeated mastery experiences are internalized into a generalized belief of “capability,” thereby strengthening self-efficacy both within digital environments and across broader contexts (Yuan et al., 2024). Second, the perceived control enhancement mechanism: individuals with higher levels of digital literacy are better equipped to identify and mitigate digital risks, such as online fraud and information leakage. This heightened sense of control over uncertainty further reinforces self-efficacy beliefs (Chen et al., 2024). The second and the third hypothesis of the study is formulated as follows:

H2: Social support is positively associated with young people's subjective wellbeing.
H3: Social support mediates the relationship between digital literacy and the subjective wellbeing of young people.

### 3.3 The mediating role of social support

Social support, as defined by Cohen and Wills (1985), refers to the emotional care, informational assistance, and instrumental aid that individuals obtain from their social networks (Cohen and Wills, 1985). Within the framework of Conservation of Resources Theory, social support is conceptualized as a conditional resource—an externally derived asset acquired through social interaction rather than an inherent personal trait—which plays a critical role in buffering stress and sustaining wellbeing.

From the perspective of relationship maintenance, individuals with higher levels of digital literacy are able to effectively utilize social media and instant communication platforms to sustain frequent and high-quality interactions with family members and close friends, thereby reinforcing the emotional support functions of strong-tie networks (Bai et al., 2025). From the perspective of relationship expansion, digital literacy enables individuals to transcend geographical constraints and establish weak-tie networks within professional communities, interest groups, and mutual-aid platforms, thereby gaining access to informational and instrumental support, such as employment opportunities and skill-sharing resources (Salehi et al., 2025). In terms of support acquisition efficiency, digitally literate individuals are more adept at articulating their needs precisely and identifying relevant sources of assistance within digital environments, which enhances the effectiveness of support mobilization.

In addition, according to the stress-buffering model, social support mitigates the adverse effects of stressful events on psychological health by providing emotional reassurance, cognitive guidance, and tangible assistance (Chang et al., 2024). Simultaneously, social support fulfills individuals' needs for belongingness and esteem, thereby directly enhancing life satisfaction. Thus, the fourth and fifth hypotheses of the study are as follows:

H4: Self-efficacy positively is positively associated with young people's subjective wellbeing.
H5: Self-efficacy mediates the relationship between digital literacy and the subjective wellbeing of young people.

### 3.4 The chain mediating role of social support and self-efficacy

Conservation of Resources Theory further posits that resources do not exist in isolation; rather, they are characterized by synergistic and progressive interrelations, whereby advantages in initial resources can trigger chain-like accumulation through resource investment processes (Da Motta Veiga et al., 2025). Within the analytical framework of the present study, this mechanism is manifested in the transmission pathway from social support to self-efficacy.

Specifically, digital literacy, as a form of personal characteristic resource, first creates the conditions for individuals to acquire social support. Individuals with higher levels of digital literacy are better able to utilize digital tools to maintain strong ties and expand weak ties, thereby obtaining emotional, informational, and instrumental support within digital communities. The accumulation of such conditional resources can, in turn, be transformed into enhancements in individuals' internal psychological resources (Siddiqi et al., 2025).

The facilitating effect of social support on self-efficacy can be understood through three mechanisms. First, the positive feedback mechanism: recognition, encouragement, and tangible assistance from others convey affirmative signals such as “you are capable” and “you are worthy of support,” which are subsequently internalized as positive self-appraisals of competence (Zhao, 2025). Second, the vicarious experience mechanism: within social support networks, individuals observe others' successful experiences and, through social comparison and cognitive inference, develop efficacy beliefs based on the perception that similar success is attainable (Warner and French, 2020). Third, the stress-buffering mechanism: social support mitigates stress-induced negative affect, thereby enabling individuals to cope with challenges with greater psychological stability, accumulate mastery experiences, and ultimately enhance self-efficacy (Serrano et al., 2024).

Accordingly, the influence of digital literacy on subjective wellbeing can be conceptualized as a chain-mediated process of “conditional resources → psychological resources.” As an initial resource, digital literacy facilitates the acquisition of richer social support; in turn, adequate social support enhances self-efficacy through positive feedback, vicarious learning, and stress buffering. The synergistic growth of these two forms of resources ultimately converges in the enhancement of subjective wellbeing. The sixth hypotheses of the study are as follows:

H6: Social support and self-efficacy jointly mediate the relationship between digital literacy and the subjective wellbeing of young people in a chain mediation pathway.

## 4 Study design

### 4.1 Research sample

This study defines individuals aged 18–44 as youth and uses this group as the research population (Timoshchenko et al., 2023). Based on research requirements and data accessibility, the survey was conducted across 27 provinces, municipalities, and autonomous regions in eastern, central, and western China, excluding Tibet. A total of 498 questionnaires were collected online via the “Wenjuanxing” platform. After excluding incomplete responses, duplicates, and questionnaires with identical answers for all items, 480 valid responses remained, yielding an effective response rate of 96.4%. Descriptive statistics of the sample characteristics are presented in Table 1.

**Table 1:** Descriptive analysis of sample characteristics.

Variable	Category	Frequency	Percentage (%)
Gender	Female	259	53.96
	Male	221	46.04
Age	18–25	184	38.30
	26–35	149	30.60
	36–44	147	31.00
Region	Eastern	174	36.20
	Central	145	30.20
	Western	161	33.50
Household registration	Rural	233	48.50
	Urban	247	51.40
Marital status	Married	227	47.30
	Unmarried	253	52.70
Occupation	Self-employed	141	29.38
	Enterprise Employee	45	9.38
	Government/Public Institution Staff	91	18.96
	Student	144	30.00
	Freelancer/Other	59	12.29
Health status	Poor	203	42.29
	Fair	118	24.58
	Average	25	5.21
	Good	87	18.13
	Healthy	47	9.79
Education level	Primary or below	165	34.50
	Junior/High School/Technical School/Vocational High School	201	41.80
	College/ Undergraduate	54	11.20
	Master/Doctoral	60	12.50

### 4.2 Variable measurement

This study primarily employed a questionnaire survey and statistical analysis to examine the relationships among digital literacy, the subjective wellbeing of young people, social support, and self-efficacy. All items were measured using a 5-point Likert scale, where 1 represents “strongly disagree” and 5 represents “strongly agree.” The final questionnaire consisted of five sections: basic demographic information, digital literacy, subjective wellbeing, social support, and self-efficacy. Measurement scales were adapted from existing studies and tailored to the target population. Detailed variable measurements and corresponding references are presented in Table 2. This article reverse scores the negative emotions in subjective wellbeing.

**Table 2:** Variable measurement and references.

Dimension		Measurement items	References
Digital literacy (DL)	Digital cognitive literacy (DCL)	XA1. I can provide examples of how digital technologies have changed my daily life and work.	Prior et al., 2016; Lin and Yu, 2025
		XA2. I am motivated to use the Internet to obtain useful information and online services.	
		XA3. Digital technologies have helped me improve my work, life, or study	
		XA4. I am willing to keep up with emerging trends in digital technologies.	
	Digital operational literacy (DOL)	XB1. I prefer using digital apps to access various services.	
		XB2. I tend to communicate with others through digital devices such as smartphones or computers.	
		XB3. I am able to download and install applications on smart devices.	
		XB4. I know how to express my ideas clearly on online platforms.	
	Digital security literacy (DSL)	XC1. I pay attention to protecting personal information when using the Internet.	
		XC2. I carefully review terms of service or privacy policies before downloading apps.	
		XC3. I know how to verify the source and authenticity of unfamiliar online information.	
		XC4. I can explain the importance of information security, data encryption, and virus protection.	
	Digital professional literacy (DPL)	XD1. I obtain profession- or discipline-related information through professional websites or academic platforms.	
		XD2. I can use at least one professional digital tool (e.g., design software) to complete study or work tasks.	
		XD3. I use online recruitment platforms or industry communities to search for career-related information or opportunities.	
		XD4. I learn new skills required for my profession through online courses or digital learning platforms.	
Subjective wellbeing (SWB)	Life satisfaction (LS)	YA1. My life is close to what I consider ideal.	Bradburn and Noll, 1969; Diener, 2000
		YA2. The conditions of my life are excellent.	
		YA3. I am satisfied with my life.	
		YA4. So far, I have achieved the things I consider important in life.	
	Positive affect (PA)	YB1. I find life interesting.	
		YB2. I feel that I am a person of worth.	
		YB3. I have many friends.	
		YB4. My life feels fulfilling.	
	Negative affect (NA)	YC1. I feel bored with life.	
		YC2. I feel useless.	
		YC3. I feel lonely.	
		YC4. I feel that life is empty.	
Social support (SS)	Support quantity (SQ)	MA1. I have many people I can rely on when I need help.	Thoits, 1985;
		MA2. People around me are willing to provide practical assistance.	Xiao, 1994
		MA3. My support network covers multiple areas, including daily life, emotional needs, and work.	
	Support quality (SQI)	MB1. My family and friends understand my concerns and troubles.	
		MB2. When I feel frustrated, the support I receive brings me comfort.	
		MB3. I find the support from people around me highly effective.	
Self-efficacy (SE)		M1. I am good at learning to use new digital products and services.	Geng and Shen, 2019
		M2. I am confident in adapting quickly to the digital information environment.	
		M3. I understand better than most people how to use digital tools effectively.	
		M4. When facing challenges in digital environments, I can always find strategies to cope with them.	

### 4.3 Research methods

To better explain how digital literacy influences subjective wellbeing, this study integrates structural equation modeling (SEM) and fuzzy-set qualitative comparative analysis (fsQCA). SEM is employed to examine the net effects of digital literacy and to test its underlying mediating mechanisms. However, its reliance on linear and symmetric assumptions limits its ability to capture the complexity of this relationship. In contrast, fsQCA complements SEM by identifying multiple configurational pathways through which different dimensions of digital literacy, in combination with psychosocial factors, jointly shape subjective wellbeing. By combining these approaches, this study not only reveals the direct and mediated effects of digital literacy but also uncovers diverse causal pathways, thereby providing a more comprehensive understanding of how digital literacy contributes to the wellbeing of young people.

## 5 Data analysis and hypothesis testing

### 5.1 Reliability and validity analysis

The reliability and validity of the questionnaire were assessed using Cronbach's α coefficients and the Kaiser-Meyer-Olkin (KMO) measure. Results are presented in Table 3. All variables exhibited Cronbach's α values greater than 0.7. The KMO value for the social support dimension was 0.765, while all other variables had KMO values above 0.8. The overall scale demonstrated a Cronbach's α of 0.917 and a KMO of 0.899, indicating high internal consistency and strong reliability for measuring constructs related to young people.

**Table 3:** Analysis of questionnaire reliability and validity.

Variable	Cronbach's α	KMO	Bartlett's test of sphericity			Number of items
			χ^2^	*df*	*p*	
DL	0.855	0.861	3,219.724	120	0	16
DCL	0.852	0.823	795.195	6	0	4
DOL	0.844	0.812	760.887	6	0	4
DSL	0.824	0.807	657.348	6	0	4
DPL	0.839	0.815	728.342	6	0	4
SS	0.792	0.765	1,146.464	15	0	6
SE	0.888	0.844	1,038.774	6	0	4
SWB	0.915	0.919	3,645.726	66	0	12
Overall scale	0.917	0.899	9,768.344	703	0	54

### 5.2 Reliability and validity analysis

Pearson correlation coefficients were calculated to examine the relationships among the first-order latent variables, including digital literacy, subjective wellbeing, social support, and self-efficacy. Focusing on first-order constructs allows for a more straightforward and interpretable assessment of the bivariate relationships among the core theoretical variables, while avoiding additional complexity associated with higher-order modeling. This approach is consistent with the objective of this study, which aims to explore the associations among the main constructs. The results are presented in Table 4. As shown, digital literacy, the subjective wellbeing of young people, social support, and self-efficacy were all significantly correlated, indicating strong interdependencies among the independent, dependent, and mediating variables.

**Table 4:** Pearson correlations among latent variables.

Variable	DL	SWB	SS	SE
DL	1			
SWB	0.415^***^	1		
SS	0.207^***^	0.378^***^	1	
SE	0.298^***^	0.513^***^	0.363^***^	1

^*^p < 0.05.

^**^p < 0.01.

^***^p < 0.001.

### 5.3 Structural equation modeling (SEM)

#### 5.3.1 Model fit analysis

A well-fitted model is a prerequisite for SEM analysis, as it accurately represents the structural relationships among variables within the sample of young people. The model fit indices for the proposed theoretical model are presented in Table 5. As shown, all indices fall within acceptable or good reference ranges, indicating that the theoretical model aligns well with the observed data.

**Table 5:** Fit indices of the SEM.

Fit index	Recommended value	Observed value	Evaluation
χ^2^	Smaller is better	756.539	–
χ^2^/*df*	< 3 good, < 5 acceptable	1.184	Good
GFI	>0.9 good, >0.8 acceptable	0.925	Good
AGFI	>0.9 good, >0.8 acceptable	0.913	Good
CFI	>0.9 good, >0.8 acceptable	0.987	Good
IFI	>0.9 good, >0.8 acceptable	0.988	Good
RMSEA	< 0.05 good, < 0.08 acceptable	0.020	Good
RMR	< 0.05 good, < 0.08 acceptable	0.052	Acceptable

#### 5.3.2 Path analysis of the SEM

The path coefficients of the structural equation model (SEM) are presented in Table 6. The results indicate that different factors exert heterogeneous effects on the subjective wellbeing of young people. Specifically, digital literacy has a significant positive impact on subjective wellbeing, supporting H1. At the dimension level, digital cognitive literacy and digital professional literacy significantly influence subjective wellbeing, whereas digital operational literacy and digital safety literacy do not, supporting H1a and H1d, but not H1b and H1c. These findings suggest that the contribution of digital literacy to wellbeing has shifted from mere “tool usage” toward “cognitive deepening” and “professional transformation,” reflecting a structural upgrading in the mechanisms through which individual wellbeing is generated in the digital society.

**Table 6:** Path coefficients of the SEM.

Hypothesized path	Unstd.	S.E.	C.R.	*P*	Std. (β)
DL → SWB	0.539	0.115	4.683	^***^	0.354
DCL → SWB	0.121	0.049	2.462	^*^	0.136
DOL → SWB	0.033	0.050	0.667	0.505	0.039
DSL → SWB	0.052	0.054	0.950	0.342	0.049
DPL → SWB	0.142	0.056	2.552	^*^	0.151
DL → SS	0.424	0.103	4.129	^***^	0.390
SS → SWB	0.457	0.142	3.210	^***^	0.326
DL → SE	0.411	0.130	3.163	^***^	0.235
SS → SE	0.756	0.160	4.715	^***^	0.469
SE → SWB	0.252	0.064	3.937	^***^	0.290

^*^p < 0.05.

^**^p < 0.01.

^***^p < 0.001.

Moreover, digital literacy positively and significantly affects social support and self-efficacy, while social support significantly enhances the subjective wellbeing of young people and self-efficacy, and self-efficacy in turn significantly promotes their subjective wellbeing, supporting H2 and H4.

### 5.4 Mediation effect analysis

Building on previous research and comparing various methodological approaches, the widely used bootstrap method was employed to test the mediation effects. Bootstrap resampling was set to 5,000 iterations to examine the indirect effects. The results are presented in Table 7.

**Table 7:** Mediation effect analysis results.

Effect type	Std.	S.E.	Z (Std./S.E.)	95% confidence interval	
				Lower	Upper
Total effect	0.918	0.141	6.511	0.679	1.225
Direct effect	0.539	0.136	3.963	0.287	0.827
Total indirect effect	0.379	0.087	4.356	0.245	0.590
ind1:DL → SS → SWB	0.194	0.090	2.156	0.074	0.430
ind2:DL → SE → SWB	0.104	0.046	2.261	0.022	0.202
ind3:DL → SS → SE → SWB	0.081	0.032	2.531	0.038	0.170

As shown, the 95% confidence intervals for both the direct and indirect effects of digital literacy on the subjective wellbeing of young people do not include zero, indicating that all effects are positive and significant. This suggests that digital literacy can directly enhance young people's subjective wellbeing and also exert indirect effects through social and psychological mechanisms.

Specifically, digital literacy significantly influences subjective wellbeing through two single mediation pathways: via social support and via self-efficacy. This indicates that, as young people improve their digital skills, they can leverage richer social connections and stronger self-beliefs to enhance their wellbeing. Moreover, digital literacy also affects subjective wellbeing through a chain mediation pathway: social support → self-efficacy. This implies that social support not only directly promotes wellbeing but also strengthens individual self-efficacy, generating additional psychological benefits. These results support H3, H5, and H6.

### 5.5 Configuration analysis

#### 5.5.1 Data calibration

Based on the theoretical analysis and empirical results presented above, this study selected digital cognitive literacy, digital operational literacy, digital safety literacy, digital professional literacy, social support, and self-efficacy as condition variables, with the subjective wellbeing of young people as the outcome variable. Calibration of these variables was conducted using fsQCA 4.0 software.

Specifically, the mean values of each variable were first calculated as reference points. Following Ragin's methodology (Ragin, 2006), 95%, 50%, and 5% of the descriptive statistics for each variable were set as the anchors for full membership, the crossover point, and full non-membership, respectively. The calibration results are presented in Table 8.

**Table 8:** Calibration anchors and descriptive statistics of variables.

Condition	Fuzzy-set calibration			Descriptive statistics			
	Full membership	Crossover	Full non-membership	Mean	SD	Min	Max
SWB	5.00	3.75	2.24	3.54	0.88	5.00	1.00
DCL	4.69	3.75	2.44	3.68	0.99	5.00	1.00
DOL	5.00	3.75	1.75	3.53	1.01	5.00	1.00
DSL	5.00	3.50	1.75	3.88	0.91	5.00	1.00
DPL	5.00	4.00	2.25	3.74	0.96	5.00	1.00
SS	5.00	3.83	2.17	3.69	0.83	5.00	1.00
SE	5.00	3.75	1.75	3.66	1.01	5.00	1.00

Considering that cases with a membership score of 0.500 are not included in the truth table analysis by the software, 0.001 was added to all scores of 0.500 after calibration to ensure a sufficient number of cases for analysis (Vis and Dul, 2016).

#### 5.5.2 Necessary condition analysis

The purpose of the necessary condition analysis (NCA) is to examine whether a single condition constitutes a necessary prerequisite for the outcome. Specifically, it tests whether the membership score of a causal condition exceeds that of the outcome variable, i.e., whether the set of the outcome variable is a subset of the causal condition. NCA identifies necessary conditions by analyzing the effect size and significance of each condition and evaluates the required threshold through bottleneck level analysis. The results are presented in Table 9.

**Table 9:** Necessary condition analysis (NCA) results for individual conditions.

Condition	Method	c-accuracy	Ceiling zone	Scope	*d*	*P*
DCL	CR	99.40%	0.063	1	0.063	0.003
	CE	100.00%	0.079	1	0.079	0.000
DOL	CR	99.00%	0.030	1	0.030	0.163
	CE	100.00%	0.034	1	0.034	0.192
DSL	CR	100.00%	0.008	1	0.008	0.890
	CE	100.00%	0.016	1	0.016	0.879
DPL	CR	98.30%	0.123	1	0.123	0.000
	CE	100.00%	0.141	1	0.141	0.000
SS	CR	99.40%	0.131	1	0.131	0.000
	CE	100.00%	0.151	1	0.151	0.000
**SE**	CR	99.00%	0.107	1	0.107	0.000
	CE	100.00%	0.130	1	0.130	0.000

The NCA results indicate that social support (*d* = 0.151), digital professional literacy (*d* = 0.123), and self-efficacy (*d* = 0.107) are significant necessary conditions for the subjective wellbeing of young people. This suggests that, in the absence of any of these three factors, young people are unlikely to achieve high levels of wellbeing. Social support reflects the structural constraints of external social capital, digital professional literacy represents a critical threshold for transforming digital skills into value creation and self-realization, and self-efficacy serves as an internal resource that supports psychological resilience and subjective wellbeing.

In contrast, digital cognitive literacy shows only weak necessity, while digital operational and safety literacy no longer constitute decisive factors for differences in wellbeing. These findings imply that the key challenges to young people's wellbeing have shifted from “basic digital skills” to “advanced digital applications and the integration of psychosocial resources,” revealing a hierarchical constraint mechanism through which digital literacy influences subjective wellbeing.

#### 5.5.3 Bottleneck level analysis of individual necessary conditions

Further analysis of the bottleneck levels for individual necessary conditions is presented in Table 10. The NCA bottleneck analysis reveals that the enhancement of the subjective wellbeing of young people exhibits distinct stage-dependent characteristics. At low levels of wellbeing, none of the conditions are necessary. Once the wellbeing level exceeds 50%, digital professional literacy and social support emerge first as constraining factors, indicating that initial improvements in wellbeing depend on the alignment of individual capabilities and environmental support.

**Table 10:** Bottleneck levels (%) of individual necessary conditions in NCA (CR method).

SWB (%)	DCL	DOL	DSL	DPL	SS	SE
0	NN	NN	NN	NN	NN	NN
10	NN	NN	NN	NN	NN	NN
20	NN	NN	NN	NN	NN	NN
30	NN	NN	NN	NN	NN	NN
40	NN	NN	NN	NN	NN	NN
50	NN	NN	NN	1.70	6.50	NN
60	NN	NN	NN	10.90	14.20	0.60
70	NN	NN	NN	20.00	21.90	13.60
80	10.00	NN	NN	29.20	29.60	26.70
90	30.40	11.30	NN	38.30	37.30	39.70
100	50.70	45.50	37.50	47.50	44.90	52.80

NN, not necessary.

As wellbeing rises further beyond 80%, self-efficacy, digital cognitive literacy, and digital safety literacy sequentially enter the necessary range, forming a multidimensional collaborative bottleneck. This suggests that achieving high levels of wellbeing requires concurrently high digital professional skills, strong psychological efficacy, and robust social support. The growth of young people's wellbeing thus demonstrates a typical “threshold effect” and “hierarchical dependency,” highlighting the stage-specific and layered nature of digital literacy and psychosocial resources in promoting subjective wellbeing.

To better examine the necessary and sufficient causal relationships in this study, we further employed the Qualitative Comparative Analysis (QCA) method to analyze the necessity of individual conditions. The results are presented in Table 11.

**Table 11:** Necessity analysis of individual conditions (fsQCA).

Causal condition	Outcome variable			
	High subjective wellbeing		Low subjective wellbeing	
	Consistency	Coverage	Consistency	Coverage
DCL	0.745	0.701	0.623	0.568
~DCL	0.541	0.596	0.672	0.719
DOL	0.736	0.703	0.605	0.561
~DOL	0.541	0.586	0.680	0.714
DSL	0.707	0.670	0.621	0.571
~DSL	0.548	0.599	0.641	0.680
DPL	0.731	0.700	0.585	0.544
~DPL	0.524	0.566	0.677	0.709
SS	0.720	0.739	0.581	0.579
~SS	0.590	0.592	0.738	0.719
SE	0.783	0.750	0.555	0.516
~SE	0.494	0.534	0.731	0.766

In fsQCA, the symbol “~” indicates the absence (negation) of a condition (e.g., “~digital cognitive literacy” refers to the absence of high levels of digital cognitive literacy).

Based on the necessity analysis results, the consistency levels of individual conditions, such as digital cognitive literacy and the absence of high levels of digital cognitive literacy, are all below 0.9, indicating that none of these conditions alone constitute a necessary condition for high subjective wellbeing. However, some differences between the NCA and fsQCA results are observed, which are theoretically and methodologically reasonable. This discrepancy primarily arises from the distinct causal logics underlying the two methods: fsQCA focuses on identifying sufficient configurations leading to high subjective wellbeing, revealing the multiple pathways through which young people can achieve wellbeing, whereas NCA is grounded in necessity logic, emphasizing which conditions act as bottlenecks whose absence constrains improvements in wellbeing.

Therefore, although the results of the two analyses are not fully consistent, they are complementary: fsQCA demonstrates multiple feasible pathways, while NCA identifies the indispensable core factors.

#### 5.5.4 Configurational analysis

For the sufficiency analysis, the consistency threshold was set at 0.8 or higher to ensure that the combinations of conditions reliably explain the outcome and to reduce the possibility of spurious associations (Oana and Schneider, 2024). In this study, the frequency threshold was set to 3, the consistency threshold to 0.9, and the PRI consistency threshold to 0.7 to eliminate causal ambiguity. Conditions appearing in both the intermediate and parsimonious solutions were considered core conditions, whereas those appearing only in the intermediate solution were considered peripheral conditions. Based on this approach, the configurations leading to high levels of the subjective wellbeing of young people were identified, as shown in Table 12. Three configurations (S1–S3) were obtained, with consistencies of 0.92, 0.93, and 0.93, respectively. The overall consistency was 0.90, exceeding the minimum standard of 0.75, indicating that all three configurations constitute sufficient conditions for enhancing young people's subjective wellbeing.

**Table 12:** Configurations for high subjective wellbeing.

Condition variables	Configurations		
	S1	S2	S3
DCL		•	•
DOL		•	○
DSL	•	•	
DPL	•		•
SS	•	•	•
SE	•	•	•
Raw Coverage	0.42	0.37	0.39
Unique Coverage	0.09	0.03	0.05
Consistency	0.92	0.93	0.93
solution coverage	0.91		
solution consistency	0.50		

“•” indicates the presence of a core condition; “○” indicates the presence of a peripheral (marginal) condition; Blank cells indicate that the presence or absence of the condition does not affect the outcome.

The configurational analysis reveals the complexity and multiplicity of pathways leading to high subjective wellbeing, consistent with the theoretical expectations of equifinality and asymmetric causality in social science research.

S1 (Security–Professional–Support Path) reflects the dual logic of “capability assurance and social embeddedness” in the digital society. Digital security literacy and professional literacy provide the foundation for stable operation and value creation in digital environments, while the simultaneous presence of social support and self-efficacy offers both external resources and internal psychological support. This enables young people to achieve a steady state of digital wellbeing in a secure and orderly network ecosystem. This pathway emphasizes that wellbeing emerges from the integration of technological, psychological, and social dimensions.

S2 (Cognitive–Operational–Social Coordination Path) shows that even without a strong professional orientation, young people can still achieve high subjective wellbeing through basic digital literacy (cognitive and operational skills) combined with social support. This indicates that wellbeing in a digital life does not fully depend on advanced technical skills, but rather on the ability to effectively integrate digital skills into social interactions and relationship-building. This pathway highlights the amplifying effect of the social dimension of digital literacy on the formation of wellbeing.

S3 (Comprehensive Capability–Driven Path) represents the “optimal combination” for wellbeing. This configuration integrates almost all dimensions of digital literacy with self-efficacy, reflecting holistic digital competence and a high degree of self-driven agency. Young people following this path can proactively adapt to digital environments, balance virtual and real interactions, and simultaneously enhance self-value and subjective wellbeing through high levels of digital professionalism and psychological self-efficacy. This mechanism demonstrates the systematic role of digital literacy in shaping subjective wellbeing: sustained improvement in wellbeing requires the synergistic resonance of technological skills, psychological motivation, and social resources.

Overall, these three pathways illustrate the multiple mechanisms through which digital literacy affects subjective wellbeing. S1 represents a structural pathway centered on professional and security literacy, S2 represents a functional pathway focused on basic operational skills and social support, and S3 represents a comprehensive pathway integrating full-spectrum literacy and psychological agency. This “multi-causal” pattern reveals that the generation of young people's digital wellbeing is not a linear causal process but a complex system driven by the interaction of multiple capabilities and resources.

## 6 Conclusion and suggestions

### 6.1 Research conclusion

This study, based on a sample of 480 young people, investigates the mechanisms through which digital literacy influences subjective wellbeing by integrating Structural Equation Modeling (SEM) and fuzzy-set Qualitative Comparative Analysis (fsQCA) within the framework of Conservation of Resources (COR) theory. The main conclusions are as follows: First, digital literacy positively influences the subjective wellbeing of young people. Dimension-level analysis shows that digital cognitive literacy and digital professional literacy have more pronounced effects, suggesting that the role of digital literacy has shifted from mere “tool usage” to “cognitive deepening” and “professional transformation,” reflecting a structural upgrading in the mechanisms underlying individual wellbeing in the digital society. Second, this effect operates through both simple and sequential mediation mechanisms: digital literacy independently enhances social support and self-efficacy, while also fostering a serial “resource–cognition” pathway in which social support further strengthens self-efficacy, ultimately promoting subjective wellbeing. Third, the configurational analysis reveals that subjective wellbeing is generated through multiple pathways, in which compensatory and synergistic mechanisms coexist, highlighting the complex and context-dependent nature of wellbeing formation. In conclusion, digital literacy promotes subjective wellbeing as a multi-layered resource system, highlighting its role as a foundational infrastructure for sustaining wellbeing in complex digital environments.

### 6.2 Policy recommendations

First, systematically integrate digital literacy education into the national education system with supporting teacher training and certification mechanisms. National education authorities should lead the development of a unified digital literacy curriculum framework and tiered competency standards covering primary, secondary, vocational, and continuing education. Concurrently, a nationwide teacher digital literacy enhancement program should be implemented, using online training platforms, school-based workshops, and summer institutes to ensure that the vast majority of in-service teachers acquire the necessary teaching competencies within a few years. Education inspectorates should include the implementation of digital literacy education in school evaluation indicators.

Second, reduce the digital access gap between urban and rural areas and among different social groups through infrastructure investment and targeted subsidies. The central government should establish a dedicated fund to support telecom operators in accelerating broadband coverage and upgrades in rural and remote areas, with clear requirements to meet basic broadband speed standards. For low-income households, migrant families, and rural youth, a regular internet subsidy mechanism (e.g., monthly fee reductions) should be established, accompanied by accessible local digital skills training. Local governments should regularly disclose network coverage and subsidy utilization data for public oversight.

Third, create structured and well-resourced opportunities for youth digital participation through government–social capital partnership models. The national development or digital economy authority should launch a dedicated program to support the establishment of local digital innovation labs, youth social enterprise incubators, and digital public welfare platforms. Projects should set measurable youth participation targets (e.g., number of participants, outputs). Through government procurement of services, tax incentives, or matching funds, technology companies, social organizations, and universities should be incentivized to participate. A substantial number of youth internships, incubation projects, or volunteer positions should be generated annually.

Fourth, strengthen platform algorithmic accountability and data privacy protection, and establish a routine regulatory mechanism for youth digital wellbeing. Cyberspace and communications regulators should require major social media and content platforms to conduct regular third-party audits, with a focus on assessing whether algorithms exacerbate filter bubbles, misinformation dissemination, and potential negative impacts on youth mental health (e.g., excessive usage, anxiety induction). Summary audit results should be made publicly available and serve as a key input for platform compliance ratings. Meanwhile, data privacy protection rules should be enhanced to clarify restrictions and disclosure obligations regarding the collection and use of youth user data. For high-risk features targeting young users (e.g., infinite scroll recommendations, personalized ad tracking), platforms should provide easy-to-access opt-out options and regularly push digital wellness reminders. Non-compliant platforms should face tiered financial penalties.

## 7 Discussion

Digital literacy has become a pivotal determinant of young people's subjective wellbeing in the digital society. By integrating Structural Equation Modeling (SEM) and fuzzy-set Qualitative Comparative Analysis (fsQCA), this study moves beyond purely linear explanations and captures both net effects and configurational complexity. More importantly, this dual-analytic approach enables a shift from viewing digital literacy as an isolated predictor to understanding it as an embedded resource within a broader causal system, thereby responding to increasing calls for incorporating causal complexity into wellbeing research.

The findings extend prior research by demonstrating that digital literacy not only exerts a direct positive effect on subjective wellbeing (He et al., 2025), but also reshapes the mechanisms through which wellbeing is generated (Li et al., 2025). The stronger effects of digital cognitive literacy and digital professional literacy suggest that digital literacy is no longer confined to functional usage, but increasingly operates as a form of higher-order cognitive and human capital resource. One possible explanation is that these advanced dimensions enhance individuals' capacity to process information, make informed decisions, and navigate complex digital environments, thereby amplifying their sense of control and competence (Ding and Zhang, 2025). This interpretation aligns with emerging perspectives that conceptualize digital literacy as a strategic resource rather than a basic skill (Zhang et al., 2024).

Moreover, the results provide deeper insights into the mediating roles of social support and self-efficacy by uncovering both independent and sequential “resource–cognition” pathways. Rather than functioning as parallel mechanisms, the sequential pathway suggests a process of resource conversion, whereby externally embedded resources such as social support are internalized into psychological resources such as self-efficacy (Jia et al., 2024). This finding resonates with resource-based perspectives, which emphasize that resources rarely operate in isolation but tend to accumulate and transform across domains. Importantly, this layered mechanism explains why digital literacy has a sustained impact on wellbeing: it not only expands access to external resources but also enhances individuals' capacity to mobilize and utilize those resources effectively (Lim et al., 2025).

From a configurational perspective, the fsQCA results further reveal that subjective wellbeing emerges through multiple equifinal pathways, highlighting the inherently complex and non-linear nature of wellbeing formation (Yang et al., 2022b). The coexistence of compensatory and synergistic configurations is particularly noteworthy. On the one hand, the compensatory patterns suggest that contextual resources, such as social support, can offset deficiencies in individual capabilities, indicating that wellbeing can still be achieved under constrained personal conditions. On the other hand, the synergistic configurations demonstrate that when multiple individual capabilities co-occur, they reinforce one another, leading to disproportionately higher levels of wellbeing (Zhang et al., 2026). This dual pattern underscores that wellbeing is not driven by single dominant factors, but by the alignment and interaction of multiple conditions within specific contexts.

Overall, this study advances the literature by offering a more nuanced and integrative framework that connects cognitive, psychological, and social dimensions in explaining subjective wellbeing. By combining SEM and fsQCA, it moves beyond linear causality and demonstrates that digital literacy influences wellbeing through both net effects and configurational pathways. More importantly, the findings highlight that the effects of digital literacy are contingent, interactive, and context-dependent, thereby challenging overly simplified models and underscoring the importance of incorporating causal complexity and heterogeneity in future research.

## 8 Limitation

First, although the theoretical model is grounded in existing literature, alternative structural relationships and path configurations may exist. Therefore, the robustness and generalizability of the model should be further tested in different contexts.

Second, the sample and cultural scope of this study are limited. The data are drawn from the Chinese context with a sample size of 480, and thus the generalizability of the findings to other cultural settings or stages of societal development requires further validation through larger-scale, cross-cultural comparative studies.

Third, the mediating mechanisms considered in this study may not be exhaustive. While social support and self-efficacy are identified as key mediators, other factors—such as digital capital, psychological resilience, and institutional trust—may also play important roles. Future research could develop more comprehensive multi-mediation models to better capture the full mechanisms through which digital literacy influences the subjective wellbeing of young people.

## Data Availability

The datasets presented in this study can be found in online repositories. The names of the repository/repositories and accession number(s) can be found in the article/supplementary material.

## References

[B1] Abah J. (2025). Digital humanitarianism and the politics of visibility: algorithmic borders, displaced bodies, and the digital agency of youth in Nigeria. J. Int. Humanitarian Action 10:16. doi: 10.1186/s41018-025-00178-9

[B2] Anderson L. (2019). Private interests in a public profession: teacher education and racial capitalism. Teach. Coll. Rec. Voice Scholarship Educ. 121, 1–38. doi: 10.1177/016146811912100602

[B3] Aydin M. (2021). Does the digital divide matter? Factors and conditions that promote ICT literacy. Telematics Inf. 58:101536. doi: 10.1016/j.tele.2020.101536

[B4] Bai X. Yu R. W. L. Liu C. Sörensen S. (2025). Digital literacy, intergenerational relationships, and future care preparation in aging chinese adults in hong kong: does the gender of adult children make a difference? Health Soc. Care Community 2025:6198111. doi: 10.1155/hsc/6198111

[B5] Bandura A. (1977). Self-efficacy: toward a unifying theory of behavioral change. Psychol. Rev. 84, 191–215. doi: 10.1037/0033-295X.84.2.191 847061

[B6] Beier E. J. Chantavarin S. Ferreira F. (2023). Do disfluencies increase with age? Evidence from a sequential corpus study of disfluencies. Psychol. Aging 38, 203–218. doi: 10.1037/pag0000741 36972092 PMC12981351

[B7] Bradburn N. M. Noll C. E. (1969). The Structure of Psychological Well-Being. Chicago, IL: Aldine Pub. Co. doi: 10.1037/t10756-000

[B8] Brown É. (2026). Democracy needs reach: political equality, online speech and algorithmic recommendation. Ethical Theory Moral Pract. doi: 10.1007/s10677-026-10535-1

[B9] Buckingham S. Tu G. Elliott L. Poole R. Walker T. Bland E. . (2023). Digital competence and psychological wellbeing in a social housing community: a repeated survey study. BMC Public Health 23:2002. doi: 10.1186/s12889-023-16875-2 37833698 PMC10576269

[B10] Burova O. (2025). Material well-being as a determinant of subjective well-being: international comparison and the Ukrainian context. Sociol. Theory Methods Mark. 126–148. doi: 10.15407/sociology2025.04.126

[B11] Carlsson N. F. (2026). Decoding digital disorientation: a conceptual framework for library engagement with adolescent mental health in a saturated media environment. J. Doc. 82, 56–78. doi: 10.1108/JD-09-2025-0276

[B12] Caroline A. Coun M. J. H. Gunawan A. Stoffers J. (2025). A systematic literature review on digital literacy, employability, and innovative work behavior: emphasizing the contextual approaches in HRM research. Front. Psychol. 15:1448555. doi: 10.3389/fpsyg.2024.1448555 39895978 PMC11783849

[B13] Castillo de Mesa J. Gómez-Jacinto L. López Peláez A. Erro-Garcés A. (2020). Social networking sites and youth transition: the use of facebook and personal well-being of social work young graduates. Front. Psychol. 11:230. doi: 10.3389/fpsyg.2020.00230 32132959 PMC7040231

[B14] Chang Y. Wang X. Liao J. Chen S. Liu X. Liu S. . (2024). Temporal hyper-connectivity and frontal hypo-connectivity within gamma band in schizophrenia: a resting state EEG study. Schizophr. Res. 264, 220–230. doi: 10.1016/j.schres.2023.12.017 38183959

[B15] Chen L. Jia J. Xiao M. Wu C. Zhang L. (2024). A study on the influence of digital literacy on elderly user's intention to identify social media false information. Electron. Library 42, 701–721. doi: 10.1108/EL-10-2023-0257

[B16] Chen X. Xiao Y. (2024). Pathways to digital reading literacy among secondary school students: a multilevel analysis using data from 31 economies. Comput. Educ. 218:105090. doi: 10.1016/j.compedu.2024.105090

[B17] Choung Y. Chatterjee S. Pak T-. Y. (2023). Digital financial literacy and financial well-being. Financ. Res. Lett. 58:104438. doi: 10.1016/j.frl.2023.104438

[B18] Cohen S. Wills T. A. (1985). Stress, social support, and the buffering hypothesis. Psychol. Bull. 98, 310–357. doi: 10.1037/0033-2909.98.2.310 3901065

[B19] Cowling M. Sim K. N. Orlando J. Hamra J. (2025). Untangling digital safety, literacy, and wellbeing in school activities for 10 to 13 year old students. Educ. Inf. Technol. 30, 941–958. doi: 10.1007/s10639-024-13183-z

[B20] Da Motta Veiga S. Debus M. Schmitz-Wilhelmy A. Ambühl M. Hasler K. Kleinmann M. (2025). Contextual and personal resources in unemployed job search: an intra-individual perspective. Appl. Psychol. 74:e12540. doi: 10.1111/apps.12540

[B21] De Vries D. A. Piotrowski J. T. de Vreese C. (2025). Developing the DigIQ: a measure of digital competence. PLoS One 20:e0322995. doi: 10.1371/journal.pone.0322995 40341757 PMC12061411

[B22] Deja M. Bobkowski P. Huvila I. Mierzecka A. (2024). Empowering through digital skills: a case of alumni in the business services sector. J. Assoc. Inf. Sci. Technol. 75, 1288–1303. doi: 10.1002/asi.24890

[B23] Deng W. Liang Q. Fan P. (2018). Social entrepreneurship and wellbeing: the configurational impact of institutions and social capital. Acad. Manag. Proc. 2018:14042. doi: 10.5465/AMBPP.2018.14042abstract

[B24] Dev M. Kumar M. Saha D. (2024). “Examining the relationship among digital inclusion of women, national cybersecurity maturity, and wellbeing: a cross-country analysis,” in Transfer, Diffusion and Adoption of Next-Generation Digital Technologies (Cham: Springer), 354–366. doi: 10.1007/978-3-031-50188-3_31

[B25] Diener E. (2000). Subjective well-being: the science of happiness and a proposal for a national index. Am. Psychol. 55, 34–43. doi: 10.1037/0003-066X.55.1.34 11392863

[B26] Diener E. Oishi S. Tay L. (2018). Advances in subjective well-being research. Nat. Hum. Behav. 2, 253–260. doi: 10.1038/s41562-018-0307-6 30936533

[B27] Ding Y. Zhang J. (2025). Does digital literacy affect the happiness of rural residents? Evidence from China. Front. Psychol. 16:1647907. doi: 10.3389/fpsyg.2025.1647907 41323912 PMC12657142

[B28] Geng R. Shen J. (2019). Research on SNS users'know ledge sharing motivation from different cultural perspectives. J. Library Sci. China 45, 60–81. doi: 10.13530/j.cnki.jlis.190005

[B29] Georgopoulou M. S. Troussas C. Krouska A. Sgouropoulou C. (2025). Digital literacy in higher education: examining university students' competence in online information practices. Computers 14:528. doi: 10.3390/computers14120528

[B30] Gilster P. (1997). Digital Literacy. New York: Wiley Computer Publishing.

[B31] Guo D. Ogbodo J. N. (2026). Bridging the digital divide: a comparative study of digital literacy and access in rural communities in China and Nigeria. Humanit. Soc. Sci. Commun. 13:243. doi: 10.1057/s41599-026-06553-0

[B32] He C. Shi R. Wen H. Chu J. (2025). Impact of digital literacy on rural residents' subjective well-being: an empirical study in China. Agriculture 15:586. doi: 10.3390/agriculture15060586

[B33] Hobfoll S. E. (1989). Conservation of resources: a new attempt at conceptualizing stress. Am. Psychol. 44, 513–524. doi: 10.1037/0003-066X.44.3.513 2648906

[B34] Huang Y. Yi D. Clark W. A. V. (2023). Subjective wellbeing in 21st century China: a multi-level multi-dimensional perspective on urban-rural disparities. Appl. Geogr. 159:103071. doi: 10.1016/j.apgeog.2023.103071

[B35] Jia W. Liu L. Peng G. (2024). The impact of social media on users' self-efficacy and loneliness: an analysis of the mediating mechanism of social support. Psychol. Res. Behav. Manag. 17, 593–612. doi: 10.2147/PRBM.S449079 38379636 PMC10876441

[B36] Kreuder A. Frick U. Rakoczy K. Schlittmeier S. J. (2024). Digital competence in adolescents and young adults: a critical analysis of concomitant variables, methodologies and intervention strategies. Humanit. Soc. Sci. Commun. 11:48. doi: 10.1057/s41599-023-02501-4

[B37] Li J. Zhou Y. Yang J. Yuan L. (2025). Digital literacy and subjective well-being among older adults: the chain mediating effect of physical exercise and consumption. J. Psychol. Afr. 35, 249–256. doi: 10.32604/jpa.2025.065790

[B38] Li S. (2025). Digital literacy and farmers' non-farm employment-an empirical study based on CFPS. Sustainable Futures 10:100999. doi: 10.1016/j.sftr.2025.100999

[B39] Li X. Su J. Mao J. Chen L. Gao H. Wang Y. . (2026). The relationship between digital literacy and mental health resilience among college students-based on the mediating role of digital learning. Front. Psychol. 17:1755407. doi: 10.3389/fpsyg.2026.1755407 41685213 PMC12891216

[B40] Lim J. H. Prihadi K. D. Tan C. Y. T. (2025). Mental health of working adults: their work and their digital literacy. Int. J. Public Health Sci. 14:594. doi: 10.11591/ijphs.v14i2.25066

[B41] Lin L. Yu J. (2025). Construction and evaluation of citizens ' digital literacy indicator system. J. Guangxi Normal Univ. 61, 79–89. doi: 10.16088/j.issn.1001-6597.2025.05.009

[B42] Lu K. Jia L. Chen S. Liang X. (2026). The impact of digital literacy on subjective well-being: evidence from China. Technol. Econ. Dev. Econ. 1–27. doi: 10.3846/tede.2026.25291

[B43] Marangell J. Randall R. (2025). Disconnected in a connected world: improving digital literacies instruction to reconnect with each other, ideas, and texts. Educ. Sci. 15:1026. doi: 10.3390/educsci15081026

[B44] Martin-Barrado A. D. Gomez-Baya D. (2025). The association between the use of digital technologies and positive youth development: a systematic review. Front. Psychol. 16:1552128. doi: 10.3389/fpsyg.2025.1552128 40688549 PMC12272609

[B45] Martini E. Sgambato M. C. (2025). Digital inequalities and access to technology: analyzing how digital tools exacerbate or mitigate social inequalities. Societies 15:318. doi: 10.3390/soc15110318

[B46] Mayen S. Reinhardt A. Wilhelm C. (2025). Revealing the interplay between digital media use and affective well-being across developmental stages: results of an experience sampling study with Austrian adolescents. J. Child. Media 19, 598–618. doi: 10.1080/17482798.2024.2443662

[B47] McKenzie R. E. Feng B. (2026). The role of website interactivity in improving depression help-seeking intentions. Patient Educ. Couns. 142:109391. doi: 10.1016/j.pec.2025.109391 41129890

[B48] Meier Y. Bol N. (2025). Unequal experiences, unequal outcomes? Digital inequalities in experiencing online benefits and privacy harms, mistrust, and self-inhibiting behaviors. J. Comp. Mediated Commun. 30:zmaf016. doi: 10.1093/jcmc/zmaf016

[B49] Narros-González M. J. Carcelén-García S. Pedreño-Santos A. (2025). Typology of young people in digital environments: identifying vulnerability patterns. Soc. Sci. Humanit. Open 12:102237. doi: 10.1016/j.ssaho.2025.102237

[B50] Ng W. (2012). Can we teach digital natives digital literacy? Comput. Educ. 59, 1065–1078. doi: 10.1016/j.compedu.2012.04.016

[B51] Nima Al A. Garcia D. Sikström S. Cloninger K. M. (2024). The ABC of happiness: validation of the tridimensional model of subjective well-being (affect, cognition, and behavior) using bifactor polytomous multidimensional item response theory. Heliyon 10:e24386. doi: 10.1016/j.heliyon.2024.e24386 38304789 PMC10831611

[B52] Oana I-. E. Schneider C. Q. (2024). A robustness test protocol for applied QCA: theory and R software application. Sociol. Methods Res. 53, 57–88. doi: 10.1177/00491241211036158

[B53] Prior D. D. Mazanov J. Meacheam D. Heaslip G. Hanson J. (2016). Attitude, digital literacy and self efficacy: flow-on effects for online learning behavior. Internet High. Educ. 29, 91–97. doi: 10.1016/j.iheduc.2016.01.001

[B54] Ragin C. C. (2006). Set relations in social research: evaluating their consistency and coverage. Polit. Anal. 14, 291–310. doi: 10.1093/pan/mpj019

[B55] Rzeszutek M. Czerwonka M. Stasiak A. Drabarek K. Van Hoy A. Pieta-Lendzion, M. . (2025). Stability of subjective well-being during the economic crisis: a four-wave latent transition analysis in a national sample of Poles. Appl. Psychol. Health Well. Being. 17:e12595. doi: 10.1111/aphw.12595 39370750

[B56] Salehi N. Nickbakht M. Branch-Smith C. Bashi N. Salehi E. Sargeant S. . (2025). Empowering social competence: a scoping review of digital social skills training interventions. Health Soc. Care Community 2025:5964176. doi: 10.1155/hsc/5964176

[B57] Serrano V. B. Pasipanodya E. C. Montoya J. L. Heaton R. K. Jeste D. V. Moore D. J. . (2024). Reactivity of health-related quality of life to perceived stress: the buffering role of psychosocial resources in a longitudinal study of adults with and without HIV. J. Clin. Psychol. Med. Settings 31, 174–185. doi: 10.1007/s10880-023-09962-4 37204645 PMC10924706

[B58] Shanmugasundaram M. Tamilarasu A. (2023). The impact of digital technology, social media, and artificial intelligence on cognitive functions: a review. Front. Cognit. 2:1203077. doi: 10.3389/fcogn.2023.1203077

[B59] Sharma R. Fantin A.-R. Prabhu N. Guan C. Dattakumar A. (2016). Digital literacy and knowledge societies: a grounded theory investigation of sustainable development. Telecomm. Policy 40, 628–643. doi: 10.1016/j.telpol.2016.05.003

[B60] Shi Q. Xu X. Zhang Y. Hu B. (2025). Research on psychological resilience, digital competence, and self-efficacy in online TCFL teachers. Behav. Sci. 15:366. doi: 10.3390/bs15030366 40150261 PMC11939549

[B61] Siddiqi K. O. Rahman Md. H. Rahman J. Radulescu M. (2025). Effect of perceived supervisor support, technological self-efficacy and technostress on nurses turnover intention: a moderated mediation model. Acta Psychol. 260:105631. doi: 10.1016/j.actpsy.2025.105631 41027065

[B62] Teng J. Cho S. (2025). Digital isolation by design: machine learning evidence of psychological harm from AI-driven social media. Res. Square doi: 10.21203/rs.3.rs-8033930/v1

[B63] Thoits P. A. (1985). “Social support and psychological well-being: theoretical possibilities,” in Social Support: Theory, Research and Applications (Dordrecht: Springer Netherlands), 51–72. doi: 10.1007/978-94-009-5115-0_4

[B64] Timoshchenko O. Ivanoshchuk D. Semaev S. Orlov P. Zorina V. Shakhtshneider E. . (2023). Diagnosis of familial hypercholesterolemia in children and young adults. Int. J. Mol. Sci. 25:314. doi: 10.3390/ijms25010314 38203485 PMC10778969

[B65] Tinmaz H. Lee Y.-T. Fanea-Ivanovici M. Baber H. (2022). A systematic review on digital literacy. Smart Learn. Environ. 9:21. doi: 10.1186/s40561-022-00204-y 40478098 PMC9175160

[B66] Vis B. Dul J. (2016). Analyzing relationships of necessity not just in kind but also in degree: complementing fsQCA with NCA. Sociol. Methods Res. 47, 872–899. doi: 10.1177/0049124115626179 30443090 PMC6195096

[B67] Warner L. M. French D. P. (2020). “Self-Efficacy Interventions,” in The Handbook of Behavior Change (Cambridge: Cambridge University Press), 461–478. doi: 10.1017/9781108677318.032

[B68] Xiao S. (1994). Theoretical basis and research application of the “Social Support Rating Scale”. J. Clin. Psychiatry 4, 98–100.

[B69] Yan R. Hu W. (2025). Does more support mean more literacy? The relationship and mechanisms between digital support and college students' digital literacy. Front. Psychol. 16:1571926. doi: 10.3389/fpsyg.2025.1571926 40337717 PMC12057485

[B70] Yang D. Zheng G. Wang H. Li M. (2022a). Education, income, and happiness: evidence from China. Front. Public Health 10:855327. doi: 10.3389/fpubh.2022.855327 35493390 PMC9039002

[B71] Yang Z. Cai X. Jiang Y. Li G. Zhao G. Wang P. . (2022b). What are the recipes of an entrepreneur's subjective well-being? A fuzzy-set approach for China. Int. J. Environ. Res. Public Health 20:417. doi: 10.3390/ijerph20010417 36612740 PMC9819742

[B72] Yasmine D. I. Colombijn F. van Deursen A. J. A. M. van Ingen E. (2025). Youth digital well-being: the role of digital skills and positive and negative digital outcomes in youth's subjective well-being. Comp. Hum. Behav. Rep. 20:100796. doi: 10.1016/j.chbr.2025.100796

[B73] Yuan X. Rehman S. Altalbe A. Rehman E. Shahiman M. A. (2024). Digital literacy as a catalyst for academic confidence: exploring the interplay between academic self-efficacy and academic procrastination among medical students. BMC Med. Educ. 24:1317. doi: 10.1186/s12909-024-06329-7 39548425 PMC11566123

[B74] Zhang J. (2025). Unveiling the mechanism of digital literacy on job quality: micro evidence from China. Cities 166:106281. doi: 10.1016/j.cities.2025.106281

[B75] Zhang J. Wang D. Ji M. Yu K. Qi M. Wang H. . (2024). Digital literacy, relative poverty, and common prosperity for rural households. Int. Rev. Financ. Anal. 96:103739. doi: 10.1016/j.irfa.2024.103739

[B76] Zhang K. de Silva R. Divigalpitiya R. P. (2026). How community-built environment and social capital are jointly associated with multidimensional health in China: a compensatory-synergy perspective. Sustainability 18:3564. doi: 10.3390/su18073564

[B77] Zhao H. (2025). The impact of social support on individual health knowledge creation in online patient communities: the mediating role of attitude. J. Knowl. Manag. 29, 937–967. doi: 10.1108/JKM-02-2024-0135

